# Micelle Formation inside Zeolites: A Critical Step in Zeolite Surfactant-Templating Observed by Raman Microspectroscopy

**DOI:** 10.1021/acsmaterialslett.1c00514

**Published:** 2021-11-29

**Authors:** Guillaume Fleury, Monica J. Mendoza-Castro, Noemi Linares, Maarten B. J. Roeffaers, Javier García-Martínez

**Affiliations:** † Centre for Membrane Separations, Adsorption, Catalysis and Spectroscopy for Sustainable Solutions (cMACS) Department of Microbial and Molecular Systems, KULeuven, Celestijnenlaan 200F, 3001 Leuven, Belgium; ‡ Laboratorio de Nanotecnología Molecular, Departamento de Química Inorgánica Universidad de Alicante, Ctra. San Vicente-Alicante s/n, Alicante E-03690, Spain

## Abstract

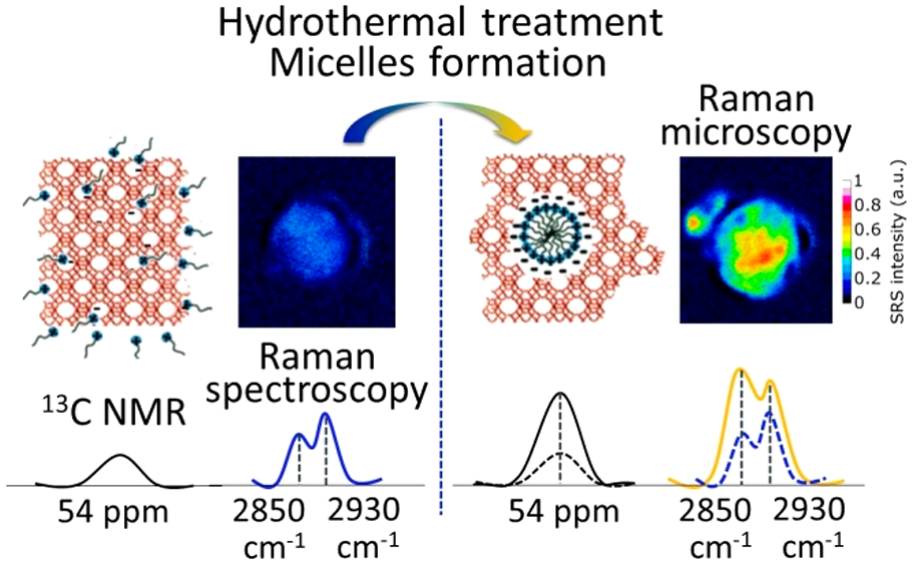

Micelle formation inside faujasite (FAU) zeolite, a critical step in the introduction of mesoporosity in zeolites by surfactant templating, has been confirmed by both ^13^C NMR and Raman spectroscopy. Here we provide unambiguous evidence of the incorporation of surfactant molecules inside zeolites during the first step of the surfactant-templating process followed by their self-assembly into micelles after hydrothermal treatment. The homogeneous presence of these micelles throughout zeolite crystals has been directly observed by Raman microspectroscopy, confirming the uniform incorporation of mesoporosity in zeolites by surfactant templating.

The development of new synthetic approaches to overcome the diffusion limitations of zeolites has been the focus of numerous studies in the past decade.^1−3^ The incorporation of a secondary mesoporous system in zeolites is a successful method for overcoming the drawbacks related to their limited accessibility, greatly enhancing their performance in the transformation of bulky molecules.^4−6^ Among the various current methods available to impart secondary porosity in zeolites,^2,3,7,8^ the postsynthetic treatment of zeolites with cationic surfactants is particularly suitable because it maintains the key features of the original zeolite such as the crystallinity, strong acidity, and hydrothermal stability.^1,9,10^

This method, known as surfactant templating, proceeds through the different steps summarized in the scheme shown in Figure 1A. First, the partial cleavage of Si–O–Si bonds by the action of a base generates negatively charged Si–O^–^ species in the zeolite framework. In a second step, the individual CTA^+^ molecules are attracted to the interior of the zeolite by the aforementioned SiO^–^ species. Finally, when the local concentration of CTA^+^ inside the zeolite is sufficiently high, micelles are formed. This causes the rearrangement of the zeolite structure to accommodate the micelles, introducing mesoporosity,^11^ which causes the expansion of the crystal.^12^ The details of this process are still elusive, but a number of techniques have been used to gain insights into the key aspects of this transformation, including its kinetics and thermodynamics, which show that surfactant templating is not only thermodynamically favorable but also kinetically accessible.^11,13^ However, one of the key steps in the surfactant templating of zeolites, namely, the formation of micelles that cause the crystal rearrangement that leads to the formation of mesopores, has never been directly confirmed. So far, only indirect evidence of micelle formation inside zeolites has been found. Thermogravimetric analysis has been used to determine the amount of CTA^+^ interacting with the crystalline structure, and small-angle X-ray diffraction and physisorption studies have been employed to confirm the templating effect of the surfactant.^11,12,14^ The diffusion of individual CTA^+^ molecules inside zeolite crystals was confirmed by repeating the same surfactant-templating method with bulkier headed surfactants (CTPA^+^), which was unable to generate any mesoporosity.^12,15^ Therefore, only small-headed surfactants (such as CTA^+^) that can diffuse through the micropores of the zeolite to reach the SiO^–^ sites are suitable for the surfactant-templating method.^12,15^

**Figure 1:**
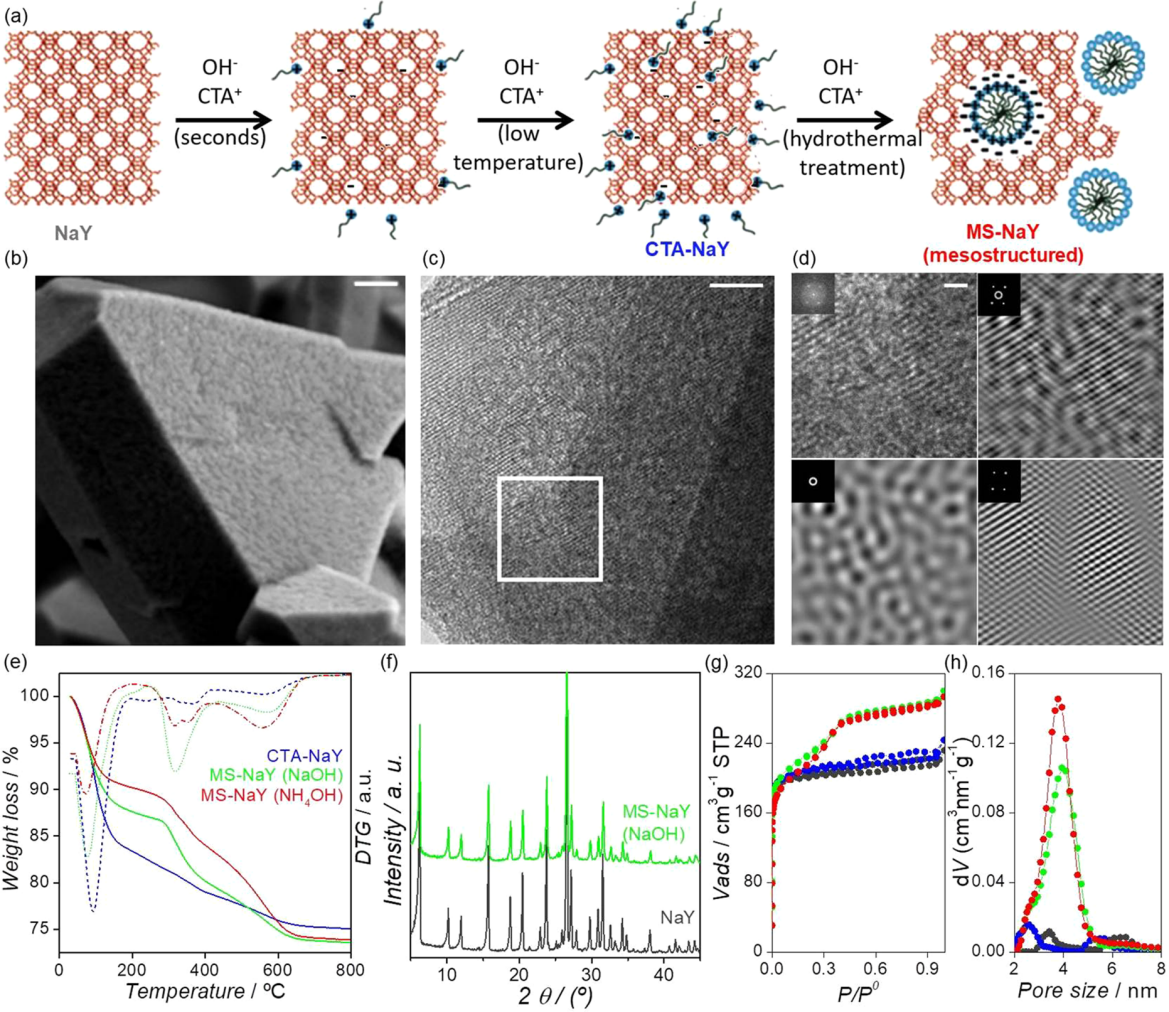
(A) Schematic representation of the surfactant-templating process in faujasite zeolite. (B) FE-SEM image of the surfactant-templated zeolite in the presence of NH_4_OH. Scale bar represents 200 nm in both images. (C) TEM micrograph of an ultramicrotomed slide of the sample MS-NaY (NaOH). Scale bar corresponds to 20 nm. The square marks the region for the fast Fourier transform (FFT) analysis. (D) Digital analysis of the TEM image. Top row: (left) Selected region for the analysis (FFT in the inset). Scale bar corresponds to 5 nm. (right) Reconstruction of the micrograph showing both mesoporosity and crystallinity features obtained from both the spots and the halo of the FFT. Bottom row: (left) Reconstruction of the mesopore features obtained from the halo of the FFT. (right) Reconstruction of the crystalline structure from the spots of the FFT. (E) Thermogravimetric/difference thermal gravimetry (TG-DTG) measurements of zeolites before the hydrothermal treatment (blue) and after (red) the treatment with NH_4_OH at 150 °C and (green) the treatment with NaOH at 80 °C. The TGA (solid line) is presented along with the DTG data (dashed line). (F) X-ray diffraction (XRD) patterns of the original NaY zeolite (gray) and the MS-NaY (NaOH). (G) N_2_ physisorption isotherms at 77 K for the original NaY (black), the pretreated NaY (blue), and the surfactant-templated zeolites with different bases, NH_4_OH (red) and NaOH (green), and (H) their corresponding pore size distribution.

Herein the use of Raman microspectroscopy allows us to go a step forward in the characterization of these surfactant-templated mesoporous zeolites. Through spatially resolved molecular spectroscopy, we have been able to see and distinguish, for the first time, not only the entry of CTA^+^ into zeolites but, what is more relevant, also the formation of micelles inside the zeolites that underwent the reported procedure. The formation of micelles inside the zeolite crystals is the key for this method, which has been a great matter of debate since it was first reported. Raman scattering is well-suited for the characterization of organic molecules and their organization.^16−19^ The vibrational frequency and the relative intensity of the Raman modes shed light on the presence of functional groups in molecules and their local environment and organization. Through Raman microscopy, these insights can be spatially resolved in 3D on the submicrometer scale. Traditional, broadband spontaneous Raman microspectroscopy is particularly suited for resolving detailed molecular information from selected locations inside complex materials.^17^ The longer acquisition time per location, typically on the second scale, however, limits mapping large volumes. More advanced, coherent Raman spectroscopy, such as stimulated Raman scattering (SRS) microscopy, is ideally suited to trace specific vibrational signatures throughout samples.^19^ As such, the combination of broadband spontaneous Raman microspectroscopy with single-band SRS microscopy mapping allows one to investigate the molecular distribution and the local environment in complex materials on the submicron scale. Moreover, the analysis of individual particles permits one to assess the homogeneity of the final material, contrarily to the possible formation of two different phases (an amorphous mesoporous material and a crystalline microporous phase) that has been reported in the past.^20,21^

Figure 1A shows the main steps involved in the surfactant templating of zeolites. Large NaY zeolite crystals were synthesized with an average size of 2000 nm (Figure 1B) so that they could be studied using optical microscopy; see the SI for details. The presence of a high Al content within the zeolite framework (Si/Al = 3.3) hinders the opening of the Si–O–Al bonds by the base (Figure S1). To overcome this limitation, a mild acid pretreatment was carried out to cleave some of the Si–O–Al bonds.^12,22,23^ Subsequently, and because of the large size of the particles, the acid-washed zeolite was stirred for 6 h in a neutral solution of surfactant to enhance the diffusion of CTA^+^ molecules inside the crystals. These two pretreatments do not produce any mesoporosity, as shown in Figure 1G,H (blue line), yet when this mixture was hydrothermally treated in the presence of a base (with either NaOH or NH_4_OH; see the SI for details), a large amount of well-defined mesoporosity develops, as shown by their N_2_ isotherms at 77 K (Figure 1G) and the field-emission–scanning electron microscopy (FE-SEM) and transmission electron microscopy (TEM) micrographs (Figure 1B–D; see Figures S2 and S3 for details of the digital analysis).

These highly mesoporous crystals maintain all of the X-ray diffraction peaks (Figure 1F) and the characteristic octahedral shape of the parent NaY (Figure 1B and Figure S2). The amount of CTA^+^ interacting with the zeolite framework was determined by thermogravimetric analysis (TGA). As previously reported,^11,24^ the fraction of surfactant that is responsible for the formation of mesoporosity corresponds to the amount of CTA^+^ removed between 250 and 400 °C. Figure 1E shows the TGA of (1) the zeolite after the pretreatments and before the hydrothermal process (CTA-NaY) and mesoporous zeolites after the hydrothermal treatment by using (2) NaOH as a base (MS-NaY (NaOH)) and (3) NH_4_OH as a base (MS-NaY (NH_4_OH)). As expected, the amount of CTA^+^ in the 250–400 °C range was higher in the hydrothermally treated sample, which is related to the formation of the micelles and, subsequently, of the mesoporosity.^12,24^

The micellization of the CTA^+^ inside the zeolite and its subsequent effect on the zeolite structure were studied by solid-state ^13^C and ^29^Si NMR; see Figure 2. The ^13^C NMR spectrum of the CTA^+^-containing zeolite after the hydrothermal treatment is shown in Figure 2A (red). For comparison purposes, the spectra of two control samples are also included, namely, the solid crystalline CTAB (light blue) and a physical mixture of NaY and CTAB (black). The physical mixture was prepared using the same NaY/CTAB ratio determined in the surfactant-templated zeolite by TGA. The chemical shifts of the ^13^C NMR spectrum of CTAB (Figure 2B, light blue) closely match those reported elsewhere.^25^ The complete assignment according to its structural formula can be found in Figure S4. The physical mixture of the zeolite and the surfactant shows very similar chemical shifts for the CTAB molecules, indicating a similar chemical environment.^26^ However, after the hydrothermal treatment, the surfactant molecules in the surfactant-templated zeolite produce broader resonance peaks, which are shifted compared with the solid CTAB. This behavior suggests a more fluid-like environment similar to a micellar solution of the surfactant. Similar findings have also been reported for the mesoporous MCM-41 silica, indicating the presence of micelles in the zeolite crystals.^27−29^ The sharp resonance at 54 ppm is characteristic of micelled (or trapped) surfactant in the zeolite structure,^28,30^ whereas the broadening and decrease in the intensity of the peak at ca. 67 ppm is due to the interaction of the surfactant heads with the polar environment inside the zeolitic structure, which changes the chemical environment of the CTAB. To study the micellization process, we analyzed the CTA-containing zeolite before the hydrothermal treatment (see Figure 2B). The obtained spectrum shows chemical shifts similar to those of the mesostructured zeolite, although the resonances are much broader and with lower intensity, even with the disappearance of some signals. This observation points out the lack of densely packed molecules, suggesting more isolated molecules within the zeolite framework.^30^ Solid-state ^29^Si NMR spectra were carried out to gain new insights into the changes in the zeolite framework caused by the surfactant-templating treatment (Figure 2C). The original zeolite shows the characteristic peaks associated with the different structural units of the aluminosilicate framework (Si(0Al), Si(1Al), Si(2Al), Si(3Al))^31^ (see Figure 2C), which are very similar to those of the physical mixture. However, in the hydrothermally treated sample, a new broad peak at a higher field appears, whereas the Si(0Al) band shifts to a lower field, which can be attributed to the formation of different types of structural defects during the surfactant templating process,^32^ which is related to the formation of the mesoporous system.

**Figure 2:**
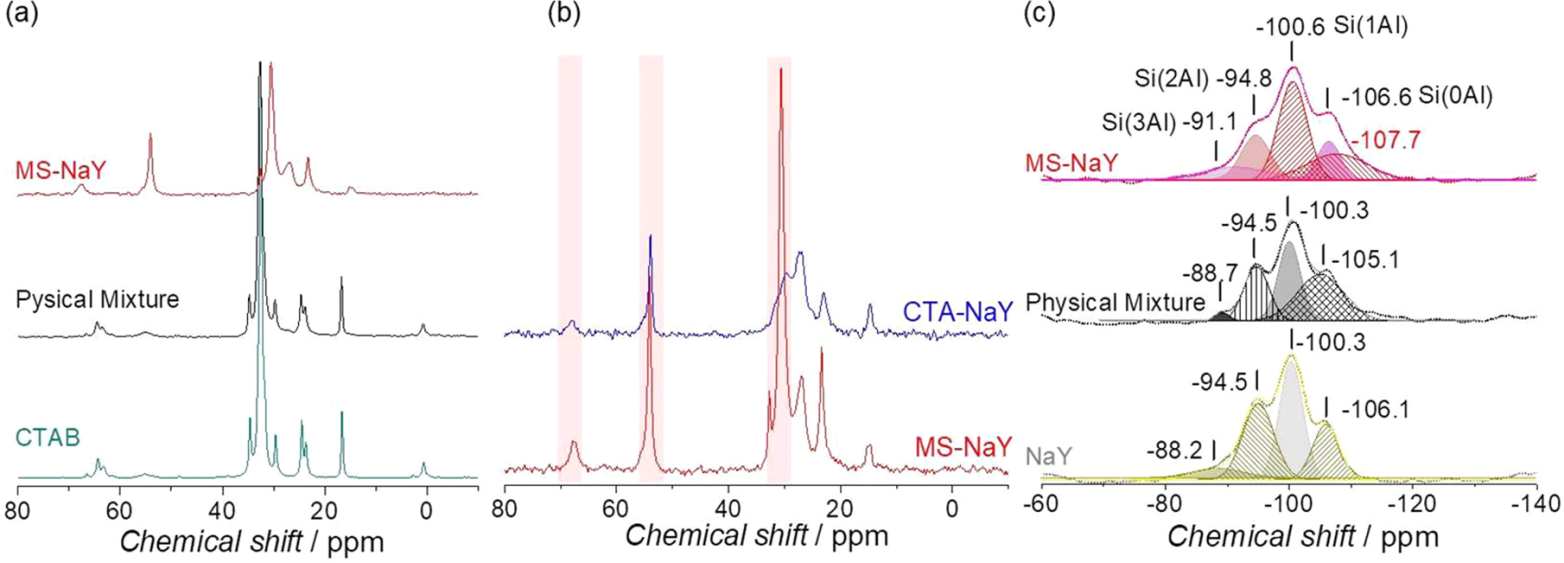
(A) Solid-state ^13^C NMR spectra of the surfactant-templated zeolite (red), a physical mixture of NaY and CTAB (black), and CTAB (light blue). (B) Solid-state ^13^C NMR of the surfactant-templated zeolite (red) and the CTA^+^-containing zeolite before the hydrothermal treatment (blue). (C) Solid-state ^29^Si spectra of the surfactant-templated zeolite (red), the original NaY (gray), and a physical mixture of NaY and CTAB (black).

These bulk-scale data suggest that the surfactant-templated mesostructuration process starts with the adsorption of isolated CTA^+^ cations inside the zeolite crystals during the pretreatment, which, upon hydrothermal treatment, restructure into micelles, leading to the formation of well-defined mesoporosity.

To gain further insights into the distribution and local organization of CTA^+^ cations, we characterized the CTAB-loaded samples at the single zeolite crystal level using spontaneous Raman microspectroscopy. First, the uptake of CTA^+^ molecules was studied using the CH-stretching vibrations of the CTA^+^ cation (2800–3100 cm^–1^) and those in relation to the zeolite framework signatures (300–600 cm^–1^) (Figure 3a). The more intense signals in the CH region after hydrothermal treatment suggest a higher CTA^+^ loading in these crystals, in agreement with the TGA data; to compare the Raman signals of CTA^+^ in crystals before and after hydrothermal treatment, we normalized the spectra with respect to the intensity of the band associated with the four-membered rings of the zeolite, centered at ca. 510 cm^–1^.^33^

**Figure 3:**
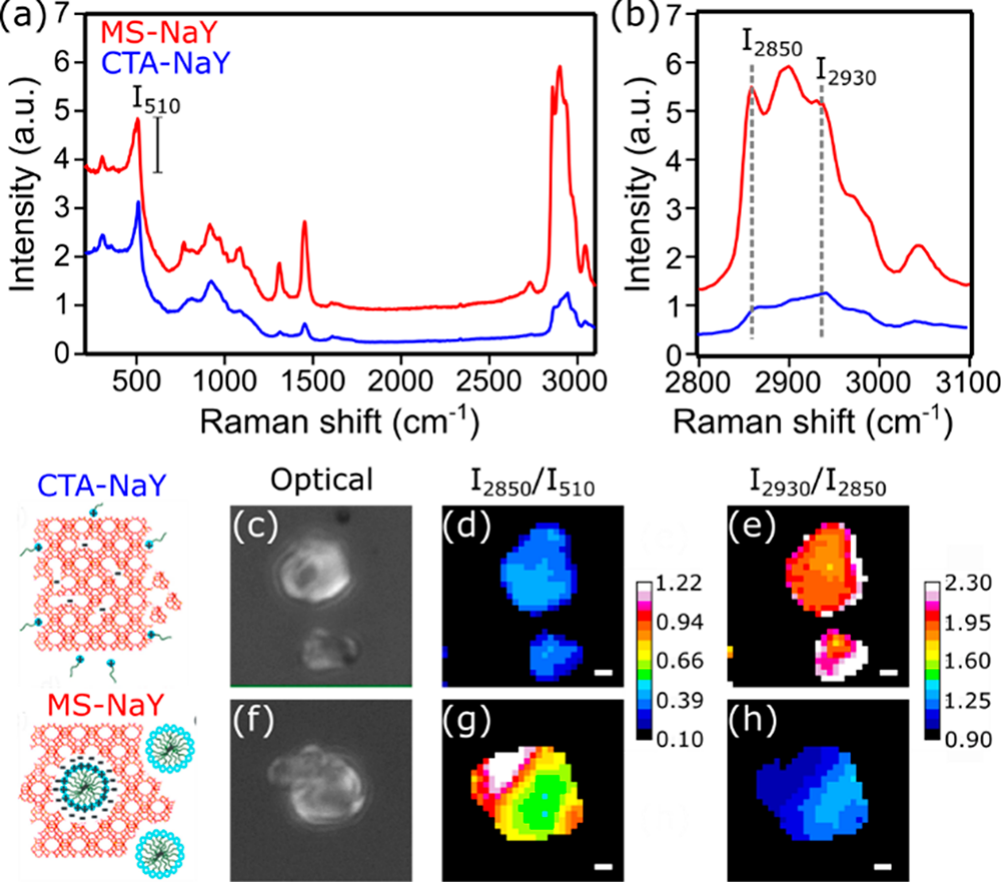
(a) Raman spectra of CTA^+^-loaded zeolite Y crystals before (blue) and after (red) hydrothermal treatment. (b) CH-stretching vibrational signatures of both samples. (c,f) Optical images of representative zeolite Y crystals before and after hydrothermal treatment, respectively. (d,g) Maps of the Raman signal in the CH-stretching region (2850 cm^–1^) normalized by the contribution of the zeolite framework (510 cm^–1^) of pretreated and hydrothermally treated crystals, respectively (scale bar: 1 μm). (e,h) Map of the ρ_2930/2850_ ratio in pretreated and hydrothermally treated crystals, respectively (scale bar: 1 μm).

Whereas these Raman spectra were collected from a location at the center of individual zeolite particles, the presence of CTA^+^ throughout the whole zeolite was confirmed by spatially mapping the CH-stretching signal at different focal planes with SRS microscopy (Figure 4). Indeed, the SRS signal associated with the CTA^+^ cations is observed at different focal planes in both pretreated and hydrothermally treated zeolite crystals. However, the SRS intensity in pretreated zeolite crystals is much lower, in agreement with the spontaneous Raman spectroscopy data (Figure 3).

**Figure 4:**
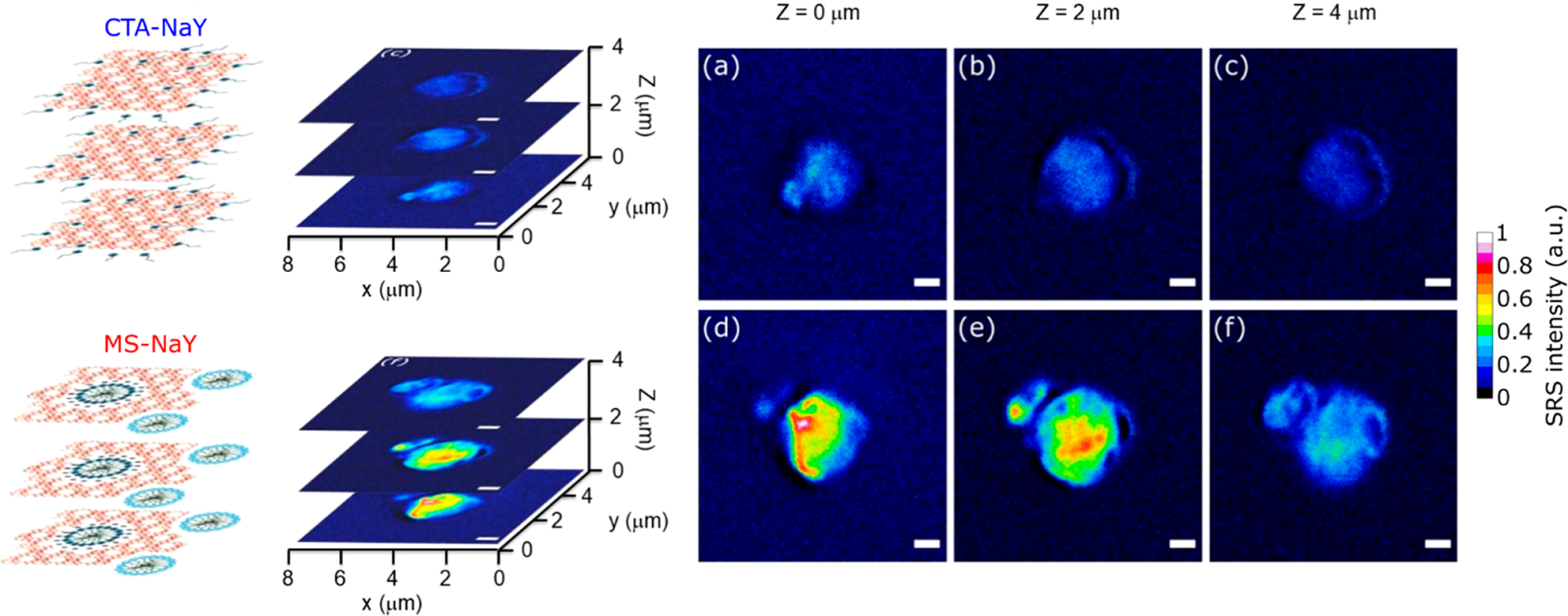
False-color SRS images acquired at 2850 cm^–1^ of zeolite Y crystals (a–c) after pretreatment and (d–f) after hydrothermal treatment at different focal planes. The SRS images were recorded at (a,d) 0, (b,e) 2, and (c,f) 4 μm from the crystal–glass interface (scale bars: 1 μm).

The CH region, while complex in nature due to the overlapping CH_2_ and CH_3_ symmetric and asymmetric stretching modes, contains valuable information on the organization of CTA^+^ cations inside the zeolite particles. Previous studies of CTAB in various solutions have linked the relative Raman signal intensity at 2930 cm^–1^ (*I*_2930_), that is the symmetric CH_3_ stretching, to that at 2850 cm^–1^ (*I*_2850_), that is the symmetric CH_2_ stretching mode, to the local polarity surrounding the CTA^+^ alkyl chain environment.^34,35^ A decreasing local polarity results in a decreased ρ_2930/2850_ = *I*_2930_/*I*_2850_. For the CTA^+^-treated zeolite Y, the ρ_2930/2850_ in crystals decreases from 1.4 before hydrothermal treatment to 0.9 after hydrothermal treatment (Figure 3a,b). This shift reveals a drastically less polar environment due to micelle formation. Note that in previous studies, micellization resulted in a similar decreasing ρ_2930/2850_. In this system, the drop in the polarity is rationalized by the transition from mostly isolated CTA^+^ cations in direct contact with the polar zeolite environment to closely packed CTA^+^ micelles with an apolar local environment. These results are in line with the observation from ^13^C NMR. The location of CTA^+^ cations in pretreated (Figure 3c) and hydrothermally treated (Figure 3f) zeolite Y crystals can be mapped by spatially resolving the Raman signature of CH-stretching modes, normalized by the signal of the zeolite framework. The hydrothermal treatment of CTA^+^-loaded zeolite Y crystals leads to a significant yet inhomogeneous increase in the CTA^+^ loading (Figure 3d,g). The heterogeneities in the surfactant molecule loading are ascribed to the nonuniform distribution of defects in the zeolite crystals. Likewise, the distribution of the ratio ρ_2930/2850_ can be assessed in individual crystals (Figure 3e,h). The local polarity of CTA^+^ cations is slightly higher in the outer shell of pretreated crystals (Figure 3e). In a similar way as the CTA^+^ distribution, the formation of micelles indicated by a decrease in the ratio ρ_2930/2850_ occurs in a nonuniform way in the hydrothermally treated crystals. Lower values of ρ_2930/2850_ are observed in intracrystalline regions with higher CTA^+^ loading.

To confirm that CTA^+^ micelle formation is not feature-specific for the large zeolite crystals used in this study, we followed the same procedure with the commercially available FAU zeolite (CBV100) typically used for mesostructuration.^12^ In a similar way, the mesostructuration of these (smaller) zeolites induced during the hydrothermal treatment also leads to a decrease in the intensity ratio of the bands centered at 2930 and 2850 cm^–1^; see Figure S5.

In summary, by combining ^13^C NMR and Raman microspectroscopy, we have been able to unequivocally prove that during the hydrothermal treatment steps, the individual CTA^+^ molecules first penetrate inside the zeolite crystals and then self-assemble to form micelles in the interior of the zeolites. Moreover, and thanks to Raman microspectroscopy, we have been able to spatially resolve in 3D the presence of these micelles throughout the zeolite crystals, which confirms our previous observations of the presence of well-defined mesoporosity homogeneously distributed inside the zeolites.

## Supporting Information Available

Experimental details regarding the synthesis and characterization of the materials and supplementary figures (showing the energy-dispersive X-ray analysis of the parent zeolite, the FE-SEM micrograph of the parent NaY zeolite, the TEM and Gatam digital analyses of the surfactant-templated zeolite, the solid-state spectrum of CTAB with the corresponding assignation, and the Raman study of the commercially available FAU zeolite) (PDF)
