# Supplementary material for: Micelle Formation inside Zeolites: A Critical Step in Zeolite Surfactant-Templating Observed by Raman Microspectroscopy
Source: ACS Mater Lett. 2021 Nov 29;4(1):49–54. doi: 10.1021/acsmaterialslett.1c00514 (PMC8729504; doi:10.1021/acsmaterialslett.1c00514)
Supplement: Supplementary file 1 — tz1c00514_si_001.pdf [file tz1c00514_si_001.pdf]

Micelles Formation inside Zeolites: A Critical Step in Zeolite Surfactant-templating Observed by Raman Microspectroscopy

Guillaume Fleury,† Monica J. Mendoza-Castro,‡ Noemi Linares,‡ Maarten B. J. Roeffaers*,† and Javier García-Martínez*,‡

† Centre for Membrane Separations, Adsorption, Catalysis and Spectroscopy for Sustainable Solutions (cMACS)
Department of Microbial and Molecular Systems
KULeuven
Celestijnenlaan 200F, 3001 Leuven, Belgium

‡ Laboratorio de Nanotecnología Molecular
Departamento de Química Inorgánica
Universidad de Alicante
Ctra. San Vicente-Alicante s/n, Alicante, E-03690 Spain

KEYWORDS. large crystals zeolites • mesoporous zeolites • surfactant-templating• micelles • Raman microspectroscopy.

ABSTRACT: Micelle formation inside FAU zeolite, a critical step in the introduction of mesoporosity in zeolites by surfactant-templating, has been confirmed by both ^{13}C NMR and Raman spectroscopy. Here we provide unambiguous evidence of the incorporation of surfactant molecules inside zeolites during the first step of the surfactant-templating process, followed by their self-assembly into micelles after hydrothermal treatment. The homogeneous presence of these micelles throughout the zeolite crystals has been directly observed by Raman microspectroscopy, confirming the uniform incorporation of mesoporosity in zeolites by surfactant-templating.

Table of Contents

1. Experimental Procedures 2

1.1 Materials 2
1.2 Sample characterization 2
1.3 Microporous NaY with large crystals preparation 2
1.4 Mesoporous zeolite preparation 2
1.5 Stimulated Raman scattering microscopy analysis 3

2. Results and Discussion 3

2.1 EDX point analysis of the original zeolite 3
2.2 FE-SEM analysis of the original zeolite 4
2.2 TEM and digital analysis of the mesoporous zeolite 4
2.2 Solid-state ^{13}C NMR spectrum of CTAB and complete assignation of each carbon 5
2.5. Spontaneous Raman microspectroscopy of smaller zeolite crystals 5

References 5

1. Experimental Procedures

1.1 Materials. Sodium aluminate (technical grade, 54 wt.% Al_2O_3, 41 wt.% Na_2O) was purchased from Riedel–de Haen. Sodium hydroxide (99%) was supplied by Merck. 15-crown-5 (98%) was purchased from Acros. Ludox AS-40 (40 wt.% SiO_2), Hexadecyltrimethylammonium bromide (98%) (CTAB), citric acid (99%) and ammonium hydroxide solution (28.0-30.0%) were purchased from Sigma-Aldrich. All reagents were used without further purification.

1.2 Sample characterization. The morphology of the mesoporous materials was investigated by transmission electron microscopy (TEM) using a JEM-2010 microscope (JEOL, 200 kV, 0.14 nm of resolution). Selected samples were embedded in a Spurr resin and cut into slices 80 nm thin using RMC-MTXL ultramicrotome (Boeckeler Instruments, Tucson, AZ). These slices were then placed on a grid to observe by TEM the cross sections of the zeolites before and after the introduction of the mesoporosity. The digital analysis of the TEM micrographs was performed using Gatam DigitalMicrograph^{TM} 1.80.70 for GMS 1.8.

Porous texture was characterized by N_2 gas adsorption at 77 K in an Quadrasorb-Kr/MP apparatus. The samples were previously degassed for 8 h at 250 ºC at 5x10^{-5} bar. Adsorption data were analyzed using the software QuadraWin^{TM} (version 6.0) of Quantachrome Instruments. Cumulative pore volumes and pore-size distribution curves were calculated using DFT method (NL-DFT adsorption branch model). The total pore volume was obtained at the plateau of the cumulative adsorption pore volume plot at a relative pressure (P/P_0) of 0.9. Micropore volume was determined by NL-DFT from the adsorption branch of the isotherms at a pore size of 2 nm, and mesopore volume was calculated by substracting the micropore volume from the total pore volume.

The Si/Al ratio of the parent NaY zeolite was determined using energy dispersive X-ray spectroscopy (EDX). The EDX spectrum of the zeolite was acquired by dispersing the powder over a conductive carbon tape before mounting the sample in a scanning electron microscope (FEI Quanta 250 FEG) equipped with an EDX detector (129 eV, 6 0mm Octane Silicon Drift Detector, EDAX). Point spectra were acquired in different regions of the sample using an acceleration voltage of 20 kV.

In order to study the surfactant incorporation and its degradation within the mesopores, thermogravimetry (TG) in combination with differential thermal analysis (DTA) experiments were carried out using a TGA/SDTA851/LF/1600 from METTLER TOLEDO at a heating rate of 10 ºC min^{-1} under a N_2:O_2 4:1 atmosphere.

MAS NMR spectra of the solids were recorded on a Bruker ADVANCE III HD 500 MHz (v^{29}Si = 99.36 MHz, v^{13}C = 125.75 MHz), with a CPMAS 4 mm zirconia probe. The following parameters and pulse sequences were used:

^{13}C CP/MAS: ^{1}H^{13}C RAMP cross-polarization. ^{1}H π/2-pulse pulse duration: 3 µs, contact time: 2 ms, relaxation delay: 3 s, number of scans: 3072. Reference: solid adamantane – 38.5 ppm (CH-group).

^{29}Si DP: Direct one pulse polarization without decoupling. π/4-pulse pulse duration: 3µs, relaxation delay: 10 s, number of scans: 576. Reference: tetramethylsilane – 0 ppm.

1.3 Microporous NaY with large crystals preparation. The zeolite Y crystals were synthesized after Delprato *et al.*,[1] by preparing a gel with a composition of 2.4 Na_2O:1.0 Al_2O_3:10 SiO_2:0.7 15-crown-5:140 H_2O. Sodium aluminate and sodium hydroxide were separately dissolved in deionized water. The sodium hydroxide solution was then added to the aluminate solution under stirring. After homogenization of the obtained gel for 2 h, Ludox AS-40 was added and the mixture was stirred for 5 minutes before adding 15-crown-5. The resulting gel was homogenized for 2 h and then transferred into a Teflon-lined autoclave. The crystallization occurred under static conditions at 110 ºC for 7 d. The product was recovered by filtration, washed with deionized water, and dried at 80 ºC for 12 h.

1.4 Mesoporous zeolite preparation. The incorporation of mesoporosity in the NaY zeolite was carried out by surfactant-templating as follows.[2–4] In a typical experiment, 1.0 g of the previously synthesized NaY was stirred in 6 mL of water while 2 mL of a 24 wt% aqueous solution of citric acid was dropwise added during one hour. The suspension was then filtered and thoroughly washed with deionized water until neutral pH. The wet cake was then suspended in a 0.22 M solution of surfactant, the mixture was heated at 60 ºC and stirred during 6 h to allow the CTA^+ to diffuse along the micropores of the zeolite. As a control, a sample was filtered and thoroughly washed with deionized water at this point, before the hydrothermal treatment. This sample was named as CTA-NaY.

For the surfactant-templated zeolites two different bases were used during the hydrothermal treatment. On one hand, 0.630 ml of a 1 M solution of NaOH was added to the abovementioned suspension and stirred during 10 min. The reaction mixture was then transferred to a Teflon® lined stainless steel autoclave,

where the hydrothermal treatment was carried out for 12 h at 80 ºC under static conditions. On the other hand, a sample prepared adding 0.920 ml of a 1 M solution of NH_4OH to the mixture and stirred during 10 min was also prepared. In this case, and due to the lower pH reached, the hydrothermal treatment was carried out at 150 ºC for 12 h. The samples were filtered, thoroughly washed with deionized water and dried at 60 ºC in an oven for 12 h. Before the textural characterization of the samples, the zeolites were calcined at 550 ºC for 6 h (2 ºC min^{-1}). The mesoporous samples were labeled as MS-NaY (NaOH) or MS-NaY (NH_4OH), respectively.

1.5 Raman scattering microscopy analysis. The spontaneous Raman spectrum of CTA^+-containing zeolite Y crystals was acquired with a confocal Raman microscope (MonoVista CRS+, S&I Instruments). The 532 nm laser line (Cobolt Samba) was used as the excitation sourced, and the laser beam was focused on the sample with a 100 × 0.9 NA objective lens (MPLN100X, Olympus). After being collected by the same objective lens, the backward Raman scattering signal was passed through a 100-μm confocal pinhole and sent into a monochromator (Princeton Instruments, Trenton, NJ, USA) equipped with a 1200 grooves/nm grating. The signal was then recorded with a charge-coupled device (CCD) camera (Newton 920, Andor). The spectrum of the sample was obtained by averaging three acquisitions of 3 s. Raman maps were obtained by scanning an area around the crystal of interest by steps of 0.25 μm in the X and Y directions.
The stimulated Raman scattering microscopy uses the pulsed (7 ps, 80 MHz) 1064 nm output of a Nd:YVO_4 laser (picoTRAIN, High-Q, Rankweil, Austria) and an optical parametric oscillator (OPO) (Levante Emerald, APE-Berlin, Germany) synchronously pumped by the 532 nm output of the of Nd:YVO_4 laser. The OPO output was wavelength tuned to probe the CH-region The amplitude of the 1064 nm laser was modulated at 9.7 MHz using a Pockell Cell (model 360-80, ConOptics, Danbury, CT, USA) and a function generator (model 29, Waveteck, Hsinchu, Taiwan) before spatial and temporal overlap with the OPO output. The SRS images were acquired in transmission on an upright optical microscope (BX61WI/FV1000, Olympus) using a water immersion 25 × 1.05 NA objective (XLPLAN, Olympus) and an oil immersion 1.4 NA condenser (U-UCD8, Olympus). Both beams had a power in focus of 45 mW. A dichroic mirror (FF750, Semrock, New York, NY, USA) was used to reflect the transmitted beams, and the 1064 nm beam was blocked with a bandpass filter (ChromaTechnology, Bellows Falls, VT, USA, CARS 890/220 m). A silicon PIN photodiode (S8650, Hamamatsu, Hamamatsu, Japan) on which a reverse bias of 60 V was applied was used to detect the stimulated Raman loss of the OPO beam. The obtained photocurrent was filtered (Mini-Circuits, Brooklyn, NY, USA, BLP-1.9), demodulated (HF2LI, Zurich Instrument, Zurich, Switzerland) and sent to an analog-to-digital converter (FV-10-ANALOG, Olympus) synchronized with the microscope.

2. Results and Discussion

2.1 EDX point analysis of the original zeolite. Figure S1 shows the typical EDX spectrum of the parent NaY zeolite before pretreatment. An average Si/Al ratio of 3.3 was determined based on 17 point measurements.

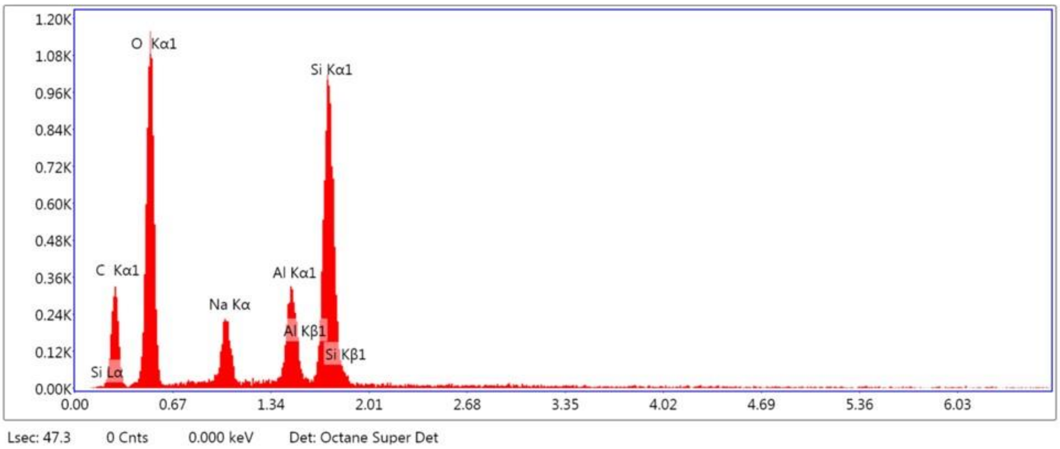

Figure S1. Typical EDX spectrum of a parent NaY crystal acquired by point analysis.

2.2 FE-SEM analysis of the original zeolite Figure S2 shows the FE-SEM image of the parent NaY zeolite before pretreatment.

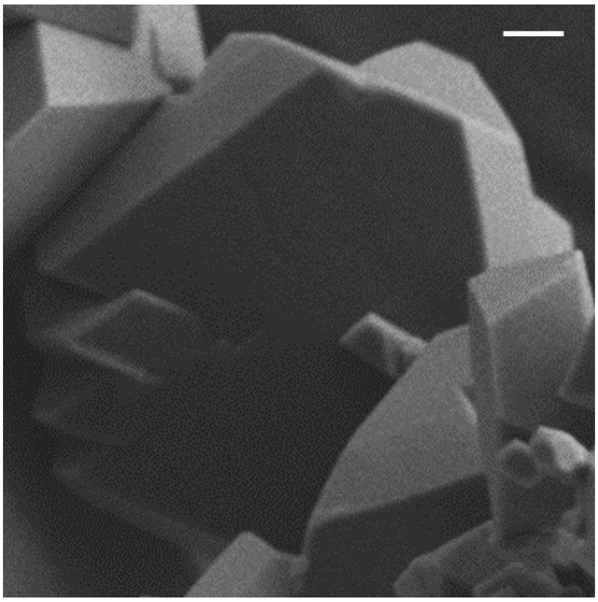

Figure S2. FE-SEM micrograph of a crystal of parent NaY zeolite. Scale bar corresponds to 20 nm.

2.3. TEM and digital analysis of the mesoporous zeolite. Figure S3 shows a TEM image of a ultramicrotomed MS-NaY (NaOH) sample and its digital analysis, which is presented here as an illustrative example. The selected area in the TEM micrograph (Figure S3b) was Fast Fourier Transformed (FFT). The achieved FTT features various spots, due to single crystal structure of the zeolite, and an inner halo, due to mesopores displaying constant pore-to-pore distance. Through masking the spots and halo reconstructed images are obtained by inversing the FFT. Figure S3d presents the reconstructed image produced only from the halo, which shows the mesopores present in the sample. Figure S3e shows the reconstructed image obtained by inversing merely the spots of the FFT, revealing the crystal lattice of the sample. By using both the spots and the halo of the FFT (inset Figure S3c), a new image was obtained, Figure S3c, which presents both features and is striking similar to the original micrograph confirming the intracrystalline nature of the mesoporosity introduced via surfactant-templating.

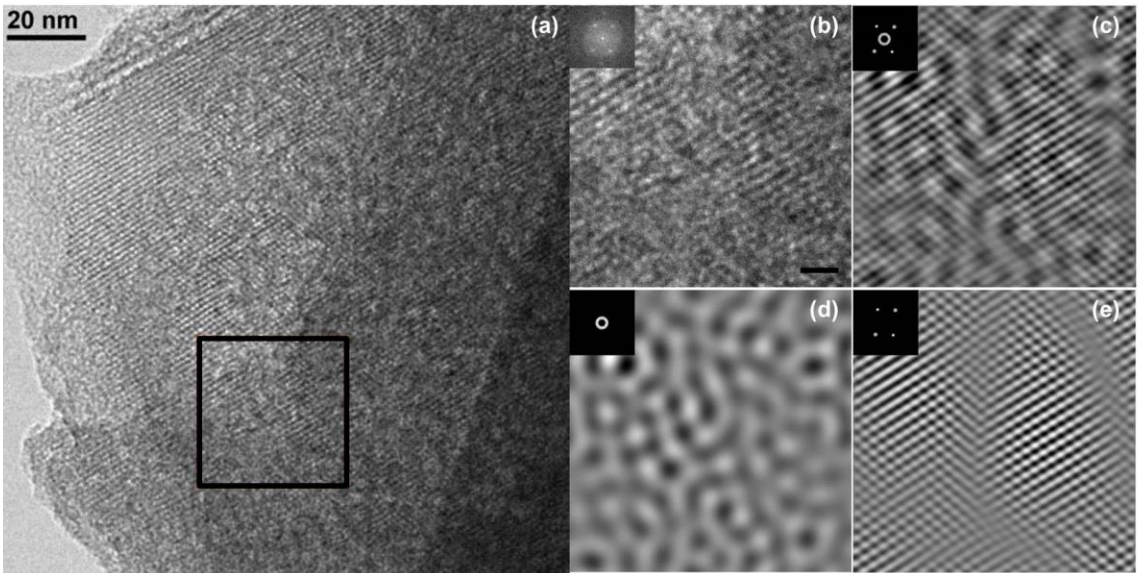

Figure S3. (a) TEM micrograph of an ultramicrotomed slide of sample MS-NaY (NaOH). Scale bar corresponds to 20 nm. (b) Selected region for the analysis (FFT in the inset). Scale bar corresponds to 5 nm. (c) Reconstruction of the micrograph showing both features mesoporosity and crystallinity, obtained from both the spots and the halo of the FFT. (d) Reconstruction of the crystalline structure from the spots of the FFT. (e) Reconstruction of the mesopore features obtained from the halo of the FFT.

2.4 Solid-state ^{13}C NMR spectrum of CTAB and complete assignation of each carbon. Figure S4 shows the solid-state ^{13}C NMR spectrum of the crystalline CTAB and the assignation of each peak related to the CTAB molecule.

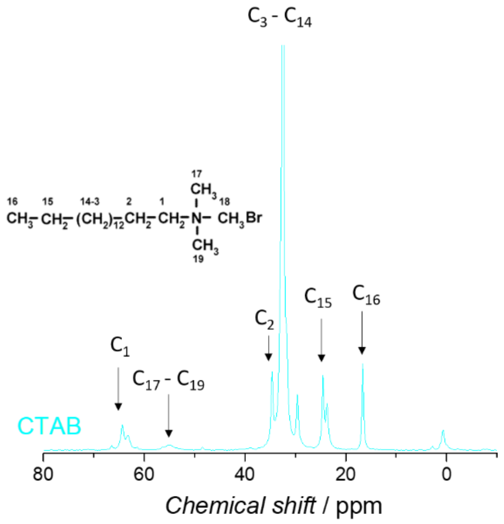

Figure S4. ^{13}C NMR spectrum of the CTAB in crystalline solid form and the assignation of the peaks.

2.5. Spontaneous Raman microspectroscopy of smaller zeolite crystals. The commercially available CBV100 was mesostructured following the procedure shown in section 1.4. The Raman microspectroscopy analysis of this zeolite before and after the hydrothermal treatment is shown in Figure S5.

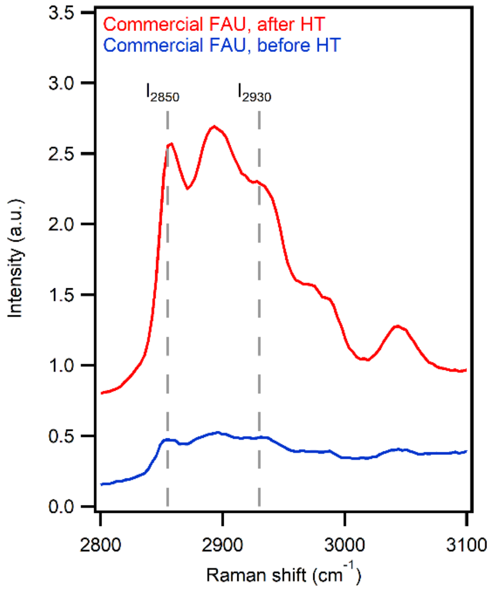

Figure S5. Raman spectra in the CH-stretching region of CTA^+-loaded commercially available zeolite Y crystals before (blue) and after (red) hydrothermally treatment.

References

[1] F. Delprato, L. Delmotte, J. L. Guth, L. Huve, *Zeolites* **1990**, *10*, 546–552.

[2] J. García-Martínez, M. Johnson, J. Valla, K. Li, J. Y. Ying, *Catal. Sci. Technol.* **2012**, *2*, 987.

[3] J. García-Martínez, K. Li, G. Krishnaiah, *Chem. Commun.* **2012**, *48*, 11841.

[4] A. Sachse, A. Grau-Atienza, E. O. Jardim, N. Linares, M. Thommes, J. García-Martínez, *Cryst. Growth Des.* **2017**, *17*, 4289–4305.
